# Epidemiological and clinical characteristics of 161 discharged cases with coronavirus disease 2019 in Shanghai, China

**DOI:** 10.1186/s12879-020-05493-7

**Published:** 2020-10-20

**Authors:** Sheng Lin, Hao Pan, Huanyu Wu, Xiao Yu, Peng Cui, Ruobing Han, Chenyan Jiang, Dechuan Kong, Yaxu Zheng, Xiaohuan Gong, Wenjia Xiao, Shenghua Mao, Bihong Jin, Yiyi Zhu, Xiaodong Sun

**Affiliations:** Shanghai Municipal Center for Disease Control and Prevention, No. 1380, West Zhongshan Road, Shanghai, 200336 China

**Keywords:** Coronavirus disease 2019, Epidemiology, Clinical characteristics, Transmission, First-level public health emergency response

## Abstract

**Background:**

In December 2019, the outbreak of coronavirus disease 2019 (COVID-19) began in Wuhan, China, and rapidly spread to other regions. We aimed to further describe the epidemiological and clinical characteristics of discharged COVID-19 cases and evaluate the public health interventions.

**Methods:**

We collected epidemiological and clinical data of all discharged COVID-19 cases as of 17 February 2020 in Shanghai. The key epidemiological distributions were estimated and outcomes were also compared between patients whose illness were before 24 January and those whose illness were after 24 January.

**Results:**

Of 161 discharged COVID-19 cases, the median age was 45 years, and 80 (49.7%) cases were male. All of the cases were categorized as clinical moderate type. The most common initial symptoms were fever (85.7%), cough (41.0%), fatigue (19.3%), muscle ache (17.4%), sputum production (14.9%), and there were six asymptomatic cases. 39 (24.2%) cases got infected in Shanghai, and three of them were second-generation cases of Shanghai native cases. The estimated median of the time from onset to first medical visit, admission, disease confirmation, and discharge for 161 cases was 1.0 day (95% CI, 0.6–1.2), 2.0 days (95% CI, 1.5–2.6), 5.2 days (95% CI, 4.6–5.7), 18.1 days (95% CI, 17.4–18.8), respectively. The estimated median of the time from admission to discharge was 14.0 days (95% CI, 13.3–14.6). The time from onset to first medical visit, admission and disease confirmation were all shortened after the Shanghai’s first-level public health emergency response. In Cox regression model, the significant independent covariates for the duration of hospitalization were age, the time from onset to admission and the first-level public health emergency response.

**Conclusions:**

Local transmission had occurred in Shanghai in late January 2020. The estimated median of the time from onset to discharge of moderate COVID-19 was 18.1 days in Shanghai. Time intervals from onset to first medical visit, admission and disease confirmation were all shortened after the Shanghai’s first-level public health emergency response. Age, the first-level public health emergency response and the time from onset to admission were the impact factors for the duration of hospitalization.

## Background

Coronavirus Disease 2019 (COVID-19) is an infectious disease caused by 2019 novel coronavirus (2019-nCoV). The most common signs of infection include fever, respiratory symptoms (such as cough and sputum production) and fatigue [1]. The first COVID-19 case was identified in Wuhan, China in late December 2019 [2]. The COVID-19 has rapidly spread from Wuhan to other areas [3, 4]. As of 17 February 2020, a total of 72,528 COVID-19 cases in China have been confirmed and cases have been reported in 25 countries and 5 continents internationally [5].

To curb the spread of COVID-19, the Shanghai authorities have declared the first-level public health emergency response on 24 January 2020 [6]. The measures included: travelers from Wuhan and other epidemic areas were advised to report their travel records and to conduct self-quarantine for 2 weeks to prevent community transmission; comprehensive implementation of sanitary quarantine at the entrance of Shanghai; cancellation various large public events; masks were recommended to be worn in public places; strengthened publicity of health knowledge, etc. Public health interventions played an important role in controlling the epidemic. As of 17 February, there were a total of 333 confirmed COVID-19 cases in Shanghai and 161 of them had been cured to discharge [7].

Epidemiological and clinical characteristics of 333 confirmed COVID-19 cases in Shanghai have been reported [8]. However, at present, the impact of first-level public health emergency response on the epidemic of COVID-19 was not estimated and information regarding the epidemiology and clinical features of discharged COVID-19 cases is scarce [9–12]. Therefore, we provided an analysis of key epidemiological determinants and clinical characteristics of 161 discharged COVID-19 cases in Shanghai. Moreover, we described and estimated the time interval from onset to discharge, which might helpful to understanding the progression of the disease.

## Methods

### Study design and participants

We performed a comprehensive study of all the 161 discharge COVID-19 cases reported in Shanghai in the case reporting system as of 17 February 2020.

### Case definition

All cases were tested COVID-19 positive in laboratory and diagnosed by clinical experts according to COVID-19 prevention and control program (4nd ed.) 2020 [13]. The symptom severity of COVID-19 was classified into moderate, severe and critical. Moderate cases refer to those cases who have symptoms such as fever and respiratory tract symptoms, etc. and pneumonia manifestations can be seen in imaging. Severe cases refer to any of the following criteria: (i) respiratory rate ≥ 30 breaths/min, (ii) oxygen saturation ≤ 93% at a rest state, (iii) arterial partial pressure of oxygen (PaO2)/oxygen concentration (FiO2) ≤ 300 mmHg. Critical cases refer to those cases that meeting any of the following criteria: (i) occurrence of respiratory failure requiring mechanical ventilation, (ii) presence of shock, (iii) other organ failure that requires monitoring and treatment in the ICU. The criteria of discharge included: (i) body temperature returned to normal (< 37.3 °C) for more than 3 days, (ii) respiratory symptoms improved significantly, (iii) rRT-PCR of 2019-nCoV was negative for two consecutive times (sampling interval at least 1 day).

### Data collection

After cases were reported to Shanghai Municipal Centers for Disease Control and Prevention (CDC), epidemiological investigations were conducted within 2 h. Demographic data, clinical symptoms or signs, laboratory tests during hospital admission, comorbidities, exposure history in 14 days and prevention and control measures were all collected. When cases were discharged, clinical records of discharge were also collected. The Specific information in epidemiological investigation was entered into a computerized database of Epidata software (Epidata Association) in duplicate. The data were analyzed anonymously.

### Laboratory testing

The 2019-nCoV laboratory test assays were based on the Technical Guidelines for Laboratory Testing of Novel Coronavirus Pneumonia [13]. Upper or lower respiratory specimens of suspected COVID-19 cases were collected and tested for 2019-nCoV by real-time reverse-transcriptase polymerase chain reaction (rRT-PCR) assay. Tests were carried out in biosafety level two facilities at district CDCs or Municipal CDC. The case was considered as laboratory tested positive only when two targets, open reading frame 1a or 1b (ORF1ab) and nucleocapsid protein (N), were both positive.

**ORF1ab:**

- Forward primer CCCTGTGGGTTTTACACTTAA;
- Reverse primer ACGATTGTGCATCAGCTGA;
- Probe 5′-VIC-CCGTCTGCGGTATGTGGAAAGGTTATGG-BHQ1–3′.

**N:**

- Forward primer GGGGAACTTCTCCTGCTAGAAT;
- Reverse primer CAGACATTTTGCTCTCAAGCTG;
- Probe 5′-FAM- TTGCTGCTGCTTGACAGATT-TAMRA-3′.

### Key points in epidemiology

The Shanghai authorities have activated the first-level public health emergency response to curb the spread of COVID-19 on 24 January. So cases were divided into two groups (illness onset during 3 Jan to 24 Jan, illness onset during 25 Jan to 17 Feb). Estimated median intervals of onset to first medical visit, onset to admission, onset to disease confirmation, admission to discharge, and onset to discharge were obtained for the two groups, assuming that the times were γ distributed.

### Statistical analysis

We present continuous variables as medians (interquartile ranges, IQR) and compared using Wilcoxon rank-sum tests between different groups. Categorical variables were described as counts and percentages in each category, and compared using chi-square or Fisher’s exact tests between different groups. Time-delay distributions (onset to first medical visit, onset to admission, onset to disease confirmation, admission to discharge, and onset to discharge) were fitted to γ distributions by maximum likelihood estimation methods. Cox regression model was used to identify factors significantly associated with the duration of COVID-19 hospitalization. These factors included: age, gender, hightest temperature, place of infection, smoking, drinking, body mass index, white blood cell count, neutrophil count, lymphocyte count, comorbidies, time from onset to admission and first-level public health emergency response.

Analyses of the time-delay distributions were performed with R software (R Foundation for Statistical Computing). Other analyses were performed with SPSS (Statistical Package for the Social Sciences) version 16.0 software (SPSS Inc).

## Results

### Demographic and clinical characteristics

**Fig. 1:**
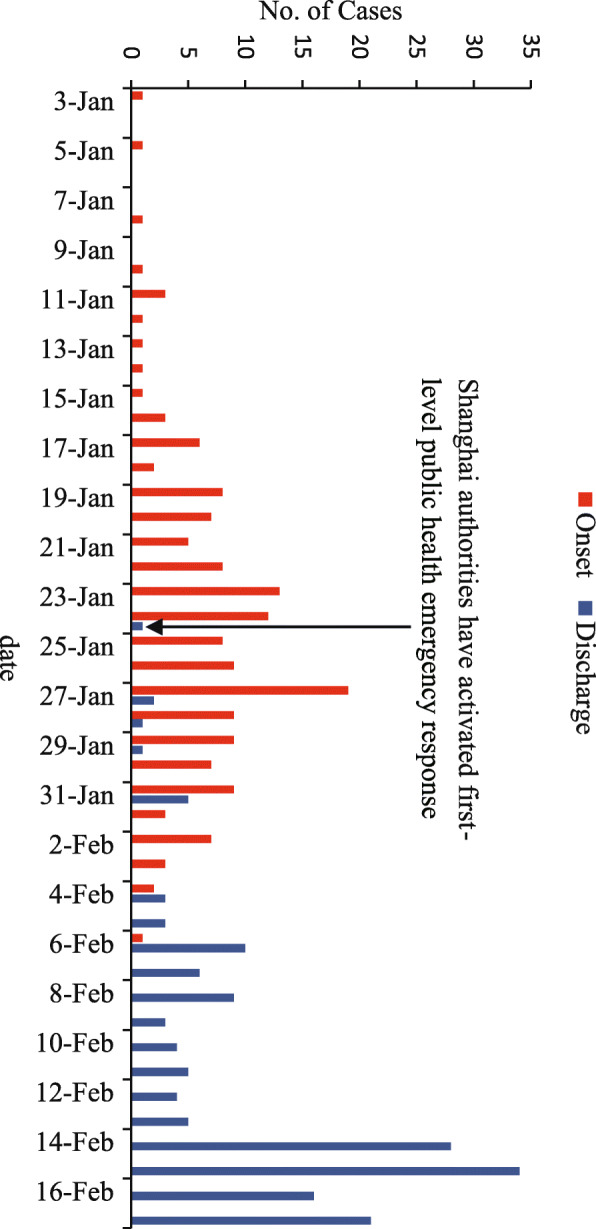
Discharge of illness among the 161 confirmed cases with COVID-19 in Shanghai, China

As of 17 February 2020, 161 confirmed COVID-19 cases had been discharged in Shanghai. The first cured case was discharged on 24 January 2020 (Fig. 1). The median age was 45 years (IQR, 34–61; range, 1–84), and four (2.5%) were younger than 15 years. 80 (49.7%) cases were male. 39 (24.2%) cases got infected in Shanghai, and three of them were second-generation cases of Shanghai native cases (Table 1).

**Table 1:** Demographic characteristics of discharged COVID-19 cases in Shanghai

Category	No. (%)			
	Overall (***N*** = 161)	Onset before 24 January (***N*** = 75) ^**a**^	Onset after 24 January (***N*** = 86)	p
Gender				0.58
Male	80 (49.7)	39 (52.0)	41 (47.7)	
Female	81 (50.3)	36 (48.0)	45 (52.3)	
Age, years				0.66
Median ([IQR])	45 (34–61)	45 (35–60)	45 (32–62)	
0–14	4 (2.5)	1 (1.3)	3 (3.5)	
15–59	113 (70.2)	54 (72.0)	59 (68.6)	
≥ 60	44 (27.3)	20 (26.7)	24 (27.9)	
Occupation				0.91
Staff in service industry	55 (34.2)	27 (36.0)	28 (32.6)	
Retiree	42 (26.1)	20 (26.7)	22 (25.6)	
Farmer/worker	9 (5.6)	5 (6.7)	4 (4.7)	
Medical staff	2 (1.2)	1 (1.3)	1 (1.2)	
Other	53 (32.9)	22 (29.3)	31 (36.0)	
Infected Place				0.02
Hubei	105 (65.2)	56 (74.7)	49 (57.0)	
Shanghai	39 (24.2)	16 (21.3)	23 (26.7)	
Other Places	17 (10.6)	3 (4.0)	14 (16.3)	
Generation				0.24
Non-Shanghai infection	122 (75.8)	59 (78.7)	63 (73.3)	
First generation	36 (22.4)	10 (13.3)	20 (23.3)	
Second generation	3 (1.9)	0 (0.0)	3 (3.5)	
Comorbidities				
Any	46 (28.6)	22 (29.3)	24 (27.9)	0.84
Hypertension	27 (16.8)	12 (16.0)	15 (17.4)	0.81
Cardiovascular disease	12 (7.5)	7 (9.3)	5 (5.8)	0.40
Diabetes	9 (5.6)	6 (8.0)	3 (3.5)	0.63
Digestive system disease	9 (5.6)	5 (6.7)	4 (4.7)	0.83
Nephropathy	4 (2.5)	3 (4.0)	1 (1.2)	0.52
Other diseases	11 (6.8)	6 (8.0)	5 (5.8)	0.58
Drinking				0.29
Yes	41 (25.5)	22 (29.3)	19 (22.1)	
No	120 (74.5)	53 (70.7)	67 (77.9)	
Smoking history				0.15
Yes	12 (7.5)	8 (10.7)	4 (4.7)	
No	149 (92.5)	67 (89.3)	82 (95.3)	
BMI				0.69
< 18·5	6 (3.7)	2 (2.7)	4 (4.7)	
18·5–23·9	92 (57.1)	45 (60.0)	47 (54.7)	
≥ 24	63 (39.1)	28 (37.3)	35 (40.7)	
Symptom severity				–
Moderate cases	161 (100.0)	75 (100.0)	86 (100.0)	
Severe cases	0 (0.0)	0 (0.0)	0 (0.0)	
Critical cases	0 (0.0)	0 (0.0)	0 (0.0)	

^a^include 24 January.

On admission, 161 were all categorized as moderate severity. The most common reported initial symptoms at illness onset were fever (138 [85.7%]) (the median highest temperature, 38.0 °C; IQR, 37.7–38.5), cough (66 [41.0%]), fatigue (31 [19.3%]), muscle ache (28 [17.4%]), sputum production (24 [14.9%]). Less common symptoms were vomit (3 [1.9%]), dyspnea (3 [1.9%]), diarrhea (5 [3.1%]) (Table 2). 107 (66.5%) cases reported fever plus any one other symptom, and 66 (41.0%) cases reported fever plus two other symptoms. 46 (28.6%) cases had one or more basic diseases, 27 (16.8%) cases had hypertension, 12 (7.5%) cases had cardiovascular diseases, 9 (5.6%) cases had diabetes.

**Table 2:** Symptoms and the first blood routine tests of COVID-19 cases since illness onset

	No. (%)			
	Overall (***N*** = 161)	Onset before 24 January (***N*** = 75) ^**a**^	Onset after 24 January (***N*** = 86)	p
Asymptomatic	6 (3.7)	0 (0.0)	6 (100)	0.06
Fever	138 (85.7)	68 (90.7)	70 (81.4)	0.09
Highest temperature, °C				
Median ([IQR])	38.0 (37.5–38.3)	38.0 (37.6–38.4)	38.0 (37.3–38.2)	0.27
< 37.3	23 (14.3)	7 (9.3)	16 (18.6)	
37.3–38.0	74 (46.0)	34 (45.3)	40 (46.5)	
38.1–39.0	59 (36.6)	32 (42.7)	27 (31.4)	
> 39.0	5 (3.1)	2 (2.7)	3 (3.5)	
Cough	66 (41.0)	36 (48.0)	30 (34.9)	0.09
Fatigue	31 (19.3)	19 (25.3)	12 (14.0)	0.07
Body aches	28 (17.4)	13 (17.3)	15 (17.4)	0.99
Sputum production	24 (14.9)	12 (16.0)	12 (14.0)	0.72
Headache	24 (14.9)	13 (17.3)	11 (12.8)	0.42
Pharyngalgia	24 (14.9)	13 (17.3)	11 (12.8)	0.42
Chill	15 (9.3)	6 (8.0)	9 (10.5)	0.59
Snivel	13 (8.1)	8 (10.7)	5 (5.8)	0.26
Nasal congestion	7 (4.3)	3 (4.0)	4 (4.7)	0.84
Loss of appetite	7 (4.3)	3 (4.0)	4 (4.7)	0.84
Chest congestion	5 (3.1)	2 (2.7)	3 (3.5)	0.76
Diarrhea	5 (3.1)	2 (2.7)	3 (3.5)	0.76
Nausea	4 (2.5)	3 (4.0)	1 (1.2)	0.52
Dyspnea	3 (1.9)	3 (4.0)	0 (0.0)	0.20
Polypnea	3 (1.9)	2 (2.7)	1 (1.2)	0.48
Vomit	3 (1.9)	3 (4.0)	0 (0.0)	0.20
Fever + at least 1 other	107 (66.5)	56 (74.7)	51 (59.3)	0.03
Fever + at least 2 other	66 (41.0)	38 (50.7)	28 (32.6)	0.04
Fever + at least 3 other	32 (19.9)	18 (24.0)	14 (16.3)	0.02
White blood cell count, × 10^9^/L (normal range 3.5–9.5)				0.45
Median ([IQR])	5.0 (3.8–6.2)	5.0 (3.7–6.0)	5.0 (4.0–6.5)	
Increased	4 (2.5)	2 (2.7)	2 (2.3)	
Normal	135 (83.9)	60 (80.0)	75 (87.2)	
Decreased	22 (13.7)	13 (17.3)	9 (10.5)	
Neutrophil count, ×10^9^/L (normal range 1.8–6.3)				0.62
Median ([IQR])	3.0 (2.3–3.9)	2.9 (2.3–3.9)	3.0 (2.3–3.9)	
Increased	9 (5.6)	3 (4.0)	6 (7.0)	
Normal	133 (82.6)	63 (84.0)	70 (81.4)	
Decreased	19 (11.8)	9 (12.0)	10 (11.6)	
Lymphocyte count, ×10^9^/L (normal range 1.1–3.2)				0.30
Median ([IQR])	1.3 (0.9–1.7)	1.2 (0.8–1.7)	1.3 (1.0–1.7)	
Increased	3 (1.9)	1 (1.3)	2 (2.3)	
Normal	93 (57.8)	39 (52.0)	54 (62.8)	
Decreased	65 (40.4)	35 (46.7)	30 (34.9)	

^a^include 24 January.

Of 161 cases, the median white blood cell counts was 5.0 × 10^9^/L (IQR, 3.8–6.2), the median neutrophil cell counts was 3.0 × 10^9^/L (IQR, 2.3–6.2), the median lymphocyte cell counts was 3.0 × 10^9^/L (IQR, 2.3–6.2).

### Key points in epidemiology

**Fig. 2:**
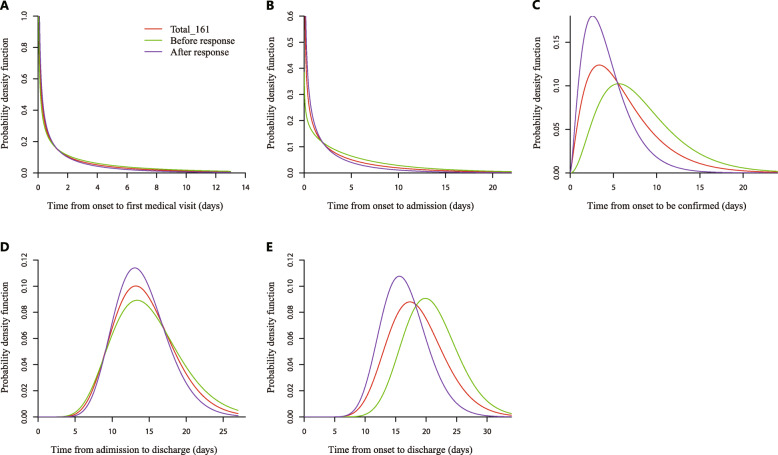
Key time to event distributions. **a** Onset to first medical visit distribution. **b** Onset to admission distribution. **c** Onset to disease confirmation distribution. **d** Admission to discharge distribution. **e** Onset to discharge distribution

The time from onset to discharge for 161 cases ranged from 7 to 34 days. The estimated median of the time from onset to discharge was 18.1 days (95% CI, 17.4–18.8) (Table 3). The estimated median of the time from onset to discharge for 75 cases who had onset symptoms before 24 January was 20.5 days (95% CI, 19.5–21.5), which was significantly longer than 86 cases with illness onset after 24 January, having a median of 16.2 days (95% CI, 15.4–17.0) (*p* < 0.001) (Fig. 2).

**Table 3:** Observed and maximum likelihood estimated time intervals

Time Intervals	Overall (***N*** = 161)		Onset before 24 January (***N*** = 75) ^**a**^		Onset after 24 January (***N*** = 86)		p
	Observed, median (IQR)	Estimated, median (95% CI)	Observed, median (IQR)	Estimated, median (95% CI)	Observed, median (IQR)	Estimated, median (95% CI)	
Onset to first medical visit (days)	2.0 (0.0–4.0)	1.0 (0.6–1.2)	2.0 (1.0–6.0)	1.7 (1.1–2.5)	1.0 (0.0–3.0)	0.6 (0.3–0.8)	< 0·001
Onset to admission (days)	3.0 (1.0–6.0)	2.0 (1.5–2.6)	5.0 (3.0–9.0)	3.7 (2.8–5.2)	2.0 (0.0–3.3)	1.1 (0.6–1.5)	< 0·001
Onset to disease confirmation (days)	5.0 (3.0–9.0)	5.2 (4.6–5.7)	8.0 (5.0–11.0)	7.3 (6.3–8.2)	4.0 (2.0–6.0)	3.7 (3.2–4.2)	< 0·001
Admission to discharge (days)	14.0 (11.0–17.0)	14.0 (13.3–14..6)	15.0 (11.0–17.0)	14.4 (13.3–15.4)	14.0 (11.0–17.0)	13.7 (12.9–14.4)	0.19
Onset to discharge (days)	19.0 (15.0–22.0)	18.1 (17.4–18.8)	21.0 (19.0–23.0)	20.5 (19.5–21.5)	17.0 (14.0–19.0)	16.2 (15.4–17.0)	< 0·001

^a^include 24 January.

The estimated median of the time from onset to first medical visit for 161 cases was 1.0 day (95% CI, 0.6–1.2). The estimated median of the time from onset to first medical visit for the 75 cases was 1.7 days (95% CI, 1.1–2.5), which was significantly longer than the 86 cases with a median of 0.6 days (95% CI, 0.3–0.8) (*p* < 0.001).

The estimated median of the time from onset to admission for 161 cases was 2.0 days (95% CI, 1.5–2.6). The estimated median of the time from onset to admission for the 75 cases was 3.7 days (95% CI, 2.8–5.2), which was significantly longer than the 86 cases with a median of 1.1 days (95% CI, 0.6–1.5) (*p* < 0.001).

The estimated median of the time from onset to disease confirmation for 161 cases was 5.2 days (95% CI, 4.6–5.7). The estimated median of the time from onset to disease confirmation for the 75 cases was 7.3 days (95% CI, 6.3–8.2), which was significantly longer than the 86 cases with a median of 3.7 days (95% CI, 3.2–4.2) (*p* < 0.001).

The estimated median of the time from admission to discharge for 161 cases was 14.0 days (95% CI, 13.3–14.6). The estimated median of the time from admission to discharge for the 75 cases was 14.4 days (95% CI, 13.3–15.4), which was similar to the 86 cases with a median of 13.7 days (95% CI, 12.9–14.4) (*p* = 0.19).

### Duration of onset to discharge and hospitalization analysis

**Fig. 3:**
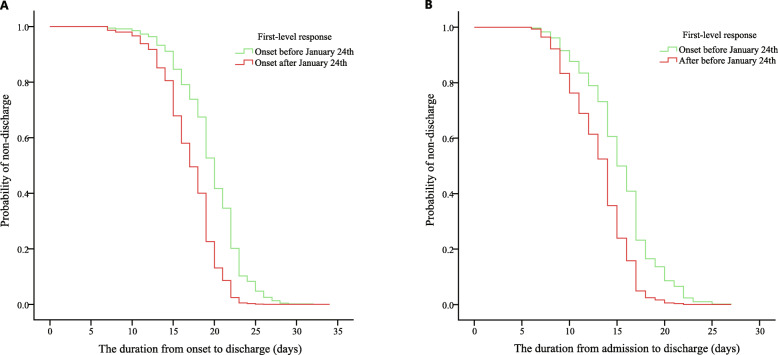
Cox regression model. **a** First-level public health emergency response impacts the duration of onset to discharge. **b** First-level public health emergency response impacts the duration of admission to discharge

In Cox regression model, we used discharge as the outcome variable (Tables 4 and 5). For all 161 cases, the significant independent covariates for the duration of onset to discharge were age, the time from onset to admission and the first-level public health emergency response. The significant independent covariates for the duration of hospitalization were age, the time from onset to admission and the first-level public health emergency response (Fig. 3). Potential influence which did not apparently impact duration of hospitalization was gender.

**Table 4:** The duration of onset to discharge analyzed by multivariate Cox regression

Factors	β	Wald χ^**2**^	***P*** Value	Exp(β) (95% CI)
First-level response				
Onset before 24 January ^a^				1.0
Onset after 24 January	0.8	18.2	< 0.001	2.3 (1.6–3.4)
Gender				
Male				1.0
Female	−0.1	0.4	0.53	0.9 (0.6–1.3)
Age	−0.01	5.3	0.02	0.98 (0.97–0.99)
Infected Place				
Shanghai		5.1	0.08	1.0
Hubei	−0.2	1.4	0.23	0.8 (0.5–1.2)
Other Places	0.3	1.2	0.27	1.4 (0.8–2.6)
Comorbidities				
No				1.0
Yes	−0.02	0.01	0.91	0.9 (0.7–1.5)
Smoking history				
No				1.0
Yes	0.4	1.5	0.22	1.5 (0.8–3.0)
Alcohol history				
No				1.0
Yes	−0.3	2.0	0.16	0.7 (0.5–1.1)
The time from onset to admission	−0.09	14.1	< 0.001	0.91 (0.87–0.96)
BMI				
Abnormal				1.0
Normal	−0.02	0.01	0.91	1.0 (0.7–1.4)
Highest temperature	−0.1	1.1	0.29	0.9 (0.7–1.1)
White blood cell count	−0.8	2.5	0.12	0.4 (0.2–1.2)
Neutrophil count	0.9	2.7	0.10	2.5 (0.8–7.7)
Lymphocyte count	0.9	1.8	0.18	2.4 (0.7–8.6)

^a^include 24 January.

**Table 5:** The duration of hospitalization analyzed by multivariate Cox regression

Factors	β	Wald χ^**2**^	***P*** Value	Exp(β) (95% CI)
First-level response				
Onset before 24 January ^a^				1.0
Onset after 24 January	0.7	13.1	< 0.001	2.1 (1.4–3.0)
Gender				
Male				1.0
Female	−0.2	1.5	0.23	0.8 (0.5–1.2)
Age	−0.02	5.6	0.02	0.98 (0.97–0.99)
Infected Place				
Shanghai		4.3	0.12	1.0
Hubei	−0.2	1.4	0.24	0.8 (0.5–1.2)
Other Places	0.3	0.9	0.35	1.3 (0.7–2.5)
Comorbidities				
No				1.0
Yes	−0.01	0.002	0.96	1.0 (0.7–1.5)
Smoking history				
No				1.0
Yes	0.4	1.1	0.29	1.4 (0.7–2.8)
Alcohol history				
No				1.0
Yes	−0.2	1.2	0.26	0.8 (0.5–1.2)
The time from onset to admission	0.1	28.9	< 0.001	1.1 (1.1–1.2)
BMI				
Abnormal				1.0
Normal	−0.1	0.1	0.74	0.9 (0.7–1.3)
Highest temperature	−0.2	1.7	0.19	0.8 (0.6–1.1)
White blood cell count	−0.9	2.8	0.09	0.4 (0.1–1.2)
Neutrophil count	1.0	3.0	0.08	2.7 (0.9–8.3)
Lymphocyte count	0.8	1.6	0.20	2.3 (0.6–8.5)

^a^ include 24 January.

## Discussion

As far as we know, this research includes the largest discharged COVID-19 case series and report an initial evaluation of the epidemiological characteristics, clinical characteristics, laboratory results, and disease course of COVID-19 cases. As of 17 February 2020, a total of 333 COVID-19 cases in Shanghai had been confirmed, of which 161 (48.3%) cases had been cured and discharged. Among them, one (0.3%) case died, and case fatality rate is consistent with national (except Hubei) [14, 15].

Among 161 discharged cases, 75.8% cases were imported to Shanghai after infection in other provinces, mainly in Hubei (65.22%). Three of them were second-generation cases of Shanghai native cases, the onset of which was late January, indicating that local transmission had occurred in Shanghai in late January. The range age of the cases was 1 to 84 years, indicating that all age groups are susceptible to the 2019-nCoV.

Common symptoms at onset of illness were fever, dry cough and fatigue. However, a significant proportion of cases presented initially with atypical symptoms, such as vomit, diarrhea and dyspnea. There were a certain proportion (14.4%) of cases without fever, if screening is focused on the detection of fever, some cases may be missed. The study found that there were six asymptomatic cases of COVID-19, which indicates that asymptomatic infections or pre-symptomatic infections is possible. The asymptomatic infections make it difficult to recognize illness and difficult to quickly and effectively isolate asymptomatic and pre-symptomatic cases, increasing the effective infectious period and the risk for transmission.

In Nanshan Chen et al. study [11], mainly in moderate patients infected with 2019-nCoV, 35% of patients had lymphocytopenia, and lymphocytopenia occurred in more than 80% of critically ill patients in Xiaobo Yang et al. study [9], indicating that the severity of lymphocytopenia reflects the severity of 2019-nCoV infection. Lymphocytopenia occurred in more than 40% of cases in our study. In the Cox regression model, the lower the lymphocyte count, the longer the duration of hospitalization, but there is no statistical significance, our sample size may be limited to finding a statistical significance.

The estimated median of the time from onset to the first medical visit, admission, disease confirmation was 1 day, 2 days and 5.2 days, respectively. After first-level public health emergency response, the time were reduced to 0.6 days, 1.1 days, and 3.7 days, respectively, which was significantly shorter than that before first-level public health emergency response (1.7 days, 3.7 days, and 7.3 days, respectively). This indicates that the early identification, isolation and confirmation of cases with COVID-19 have been accelerated after the first-level public health emergency response. Shortening the duration of onset to admission does not seem to impact clinical outcomes [16, 17]. However, shortening the duration of onset to admission facilitates quarantine and reduces the risk of transmission, and the effective communicable period. And any additional shortening of the duration that symptomatic cases are in the community will bring about further benefits at the whole crowd level.

Quarantine is a traditional and yet the most effective measure to control an epidemic. Because there is no specific vaccine or cure against 2019-nCoV infections, standard public health emergency measures usually prove most efficient, including isolating the sources of infection, interrupting or cutting off transmission routes, and special care for the most susceptible people. And the COVID-19 epidemic has shown that the essential for risk disclosure that will warn and inform the citizens, in such a way that will enhance personal protection, without triggering raised fear and anxiety, as an essential part of COVID-19 epidemic control. A change in disease risk awareness would potentially bring about an increase in early reporting of COVID-19.

Cox regression analysis showed that the elder the case, the longer the duration of hospitalization. The possible explanations were that the younger case has higher recovery ability after infection with 2019-nCoV, and the elder case has a higher proportion of comorbidities. We review previous studies that found a greater number of male than female [11, 18, 19], but our research shows that there was no significant difference in the course of disease between male and female. After first-level public health emergency response, the duration of hospitalization was shorter. Shortening the time from initial symptoms to admission does not decrease the duration of hospitalization for moderate COVID-19 cases. More generally, the average time from onset to discharge was 19 days. One reason may be that there is no specific cure or vaccine against 2019-nCoV infections except for meticulous supportive. Another reason may be that it may indicates that moderate COVID-19 is self-limited disease.

This study has several limitations. First, only three of 161 cases had short and defined periods of exposure to known COVID-19 cases, so we did not estimate the distribution of the incubation period, the time from infection to the onset of symptoms of COVID-19. Second, the symptom severity of the discharged cases was moderate pneumonia, so we are unable to estimate severe or critical pneumonia.

## Conclusions

In conclusion, local transmission had occurred in Shanghai in late January 2020. The estimated median of the time from onset to discharge of moderate COVID-19 was 18.1 days in Shanghai. Time intervals from onset to first medical visit, admission and disease confirmation were all shortened after Shanghai’s first-level public health emergency response. Age, first-level public health emergency response and the time from onset to admission were the impact factors for the duration of hospitalization. Male and female have the same course of disease.

## Data Availability

The datasets used and/or analysed during the current study are available from the corresponding author (Xiaodong Sun, sunxiaodong_scdc@163.com) on reasonable request.

## Abbreviations

COVID-19: Coronavirus Disease 2019
2019-nCoV: 2019 novel coronavirus
CDC: Centers for Disease Control and Prevention
rRT-PCR: Real-time reverse-transcriptase polymerase chain reaction
